# Knowledge, Practice, and Associated Factors towards Prevention of Surgical Site Infection among Nurses Working in Amhara Regional State Referral Hospitals, Northwest Ethiopia

**DOI:** 10.1155/2015/736175

**Published:** 2015-12-15

**Authors:** Freahiywot Aklew Teshager, Eshetu Haileselassie Engeda, Workie Zemene Worku

**Affiliations:** ^1^Gondar University Referral Hospital, University of Gondar, P.O. Box 196, Gondar, Ethiopia; ^2^Department of Nursing, College of Medicine and Health Sciences, University of Gondar, P.O. Box 196, Gondar, Ethiopia

## Abstract

Knowledge and practice of nurses about surgical site infections (SSIs) are not well studied in Ethiopia. This paper contains findings about Northwest Ethiopian nurses' knowledge and practice regarding the prevention of SSIs. The main objective of the study was to assess knowledge, practice, and associated factors of nurses towards the prevention of SSIs. The study was done using a questionnaire survey on randomly selected 423 nurses who were working in referral hospitals during the study period. The study showed that more than half of the nurses who participated in the survey had inadequate knowledge about the prevention of SSIs. Moreover, more than half of them were practicing inappropriately. The most important associated factors include lack of training on evidence based guidelines and sociodemographic variables (age, year of service, educational status, etc.). Training of nurses with the up-to-date SSIs guidelines is recommended.

## 1. Background

Worldwide, healthcare associated infections (HAIs) constitute a major public health problem affecting millions of people every year [1]. Estimates of recent studies in developed countries indicated that at least 5% of hospitalized patients acquire infection [2]. Although the burden of HAIs in Africa is not well studied, a systematic review in the region reported that it is frequently much higher than in the developed nations [1, 3]. Most literatures revealed that surgical site infections (SSIs) were the most common healthcare associated infections accounting for more than 30% of cases of HAIs [4, 5].

Surgical site infections are defined as infections that occur within 30 days of the operation if no implant is left in place or within 1 year of operation if an implant is left in place and the infection appears to be related to the operation in general surgery [6]. A systematic review in Korea reported up to 9.7% incidence rate of SSIs [7] and an Ethiopian study on patients following obstetric surgery revealed a higher rate of 11.4% [8]. Throughout the literature, SSIs were associated with intrinsic factors including advanced age, malnutrition, metabolic diseases, smoking, obesity, hypoxia, immune-suppression, and length of preoperative stay [9]. Moreover, extrinsic factors like application of skin antiseptics, preoperative shaving, antibiotic prophylaxis, preoperative skin preparation, inadequate sterilization of instruments, surgical drains, surgical hand scrubs, and dressing techniques were among the most frequently reported risk factors [10, 11].

Nurses, working around the clock, are in an ideal position to participate or play a leading role in taking initiatives that aimed to ensure quality of care and thus to enhance patient safety which includes prevention of SSIs [12]. However, a significant number of studies indicated that most nurses lacked the required knowledge about prevention of SSIs and the majority of them did not practice properly according to evidence-based guidelines and recommendations [13, 14].

According to the literature, factors associated with knowledge and practice of nurses towards the prevention of SSIs include but are not limited to work experience, level of nursing education, work load, training on infection prevention mechanisms, and nonadherence in infection prevention and patient safety guidelines [15, 16]. In some studies, insufficient utilization of available evidences was also observed [17].

The World Health Organization (WHO), in its guideline for safe surgery, has set a number of recommendations regarding the prevention of SSIs. According to this guideline, highly recommended practices to prevent SSIs include routine use of prophylactic antibiotic within 60 minutes prior to skin incision, the use of sterility indicators during sterilization of surgical instruments, presurgical skin disinfection, and the implementation of surgical safety checklist [18].

Despite the availability of some studies in the developed countries, evidences regarding the level of knowledge and practice towards prevention of SSIs and associated factors are very limited in Africa. A systematic review on SSIs in the region has also revealed that there is a critical shortage of evidences particularly in Sub-Saharan Africa including Ethiopia [3]. Therefore, this study was aimed at assessing knowledge, practice, and associated factors regarding prevention of surgical site infection among nurses working in two selected hospitals in Amhara Regional State, Northwest Ethiopia.

## 2. Materials and Methods

Institution-based cross-sectional study was conducted from March 10 to 25, 2015, in two randomly selected referral hospitals (Gondar University Referral Hospital and Debre Markos Referral Hospital) of Amhara regional State, Ethiopia. Gondar University Referral Hospital is found in Gondar Town 748 km far from Addis Ababa to the Northwest of Ethiopia. It is a teaching hospital which acts as a referral center for the nearby general hospitals. Having more than 500 inpatient beds, it provides referral services for over 5 million inhabitants in the northwest region of Ethiopia. Debre Markos Referral Hospital is also among the study sites, which is found in Debre Markos Town 305 km far from Addis Ababa to the Northwest of Ethiopia. This hospital has 400 inpatient beds and acts as a referral center for general hospitals in the area and serves 5 million inhabitants.

Sample size was determined by using single population proportion formula by considering the following assumptions: 95% confidence interval (CI), 50% proportion (since there was no previous study in the study areas), and 5% marginal error. By adding 10% nonresponse rate, the final sample size was 423. Since the study hospitals followed a yearly rotation policy (interdepartmental rotation), all nurses of the two hospitals were included in the study regardless of their working area. Simple random sampling technique was used to select the study participants. The samples were proportionally allocated to each hospital and respondents were selected using computer generated random number.

The outcome measures of this study were knowledge (knowledgeable/not knowledgeable) and practice (good practice/not good practice) of nurses regarding prevention of surgical site infection. The independent variables included sociodemographic characteristics (age, sex, marital status, religion, ethnicity, level of education, and work experience) and institutional factors (training about infection prevention, availability of antiseptic solution, availability of antibiotic prophylaxis, and availability of personal protective equipment).

English version of structured and pretested self-administered questionnaire was used to collect the data (English is the medium of instruction in all Ethiopian nursing schools). Nurse's knowledge regarding the prevention of SSIs was measured by 12 multiple choice questions in which only one correct answer was found. The questions addressed the most important recommendations by the national infection prevention and patient safety guideline (about correct time of prophylactic antibiotics, presurgical skin preparation, techniques of surgical wound dressing, etc.). On the other hand, nurses' practice in the prevention of SSIs was measured by 12 items in which responses were answered in a 3-point Likert scale (never practice, sometimes practice, and always practice).

Four diploma holders for data collection (two for each hospital) and two B.S. holders for supervision (one for each hospital) were recruited during the data collection period (both the data collectors and the supervisors were not from the same hospitals). At each hospital the aim of the study was clearly explained to the study participants before they filled the questionnaire. The data collectors and supervisors were trained for one day on how to facilitate the data collection process and prevent errors. Questionnaires were reviewed and checked for completeness, accuracy, and consistency by supervisors and the research team every day during the data collection period.

The data were coded, entered into Epi Info version 3.5.3 statistical package, and exported to SPSS version 20 software for analysis. At the beginning of the analysis, summation of the practice scale was made. Then, the variable was recoded and dichotomized. Descriptive statistics were used to illustrate the means, standard deviations, medians, and frequencies of the study variables. Bivariate analysis was computed and those variables whose *P* values are less than or equal to 0.2 were fitted into the backward stepwise multivariate logistic regression model. Odds ratios with 95% confidence interval were used to determine the strength of association between dependent and independent variables. *P* values less than or equal to 0.05 were considered as statistically significant.

Ethical clearance was obtained from University of Gondar Ethical Review Board. The aim of the study was clearly explained to participants and respected hospital officials. The data collection was begun after obtaining consent from each participant. Confidentiality was maintained by excluding the name of participants from questionnaires. No other person except the data collection facilitators and the research team members had access to filled questionnaires.

## 3. Results

### 3.1. Sociodemographic Characteristics of the Study Participants

A total of 423 nurses, 317 (74.9%) from University of Gondar Hospital and 106 (25.1%) from Debre Markos Hospital, participated in this study which made the response rate 100%. Two hundred thirty-nine (56.5%) of them were males. The median age of the study participants was 27 years with interquartile range (IQR) of 25–29 years. The majority of them (89.4%) were followers of Orthodox Christianity and B.S. degree holders (89.6%). More than half of the participants (57.8%) were singles (Table 1).

### 3.2. Knowledge about Prevention of Surgical Site Infection

The mean score of the knowledge questions was 6.19 (SD = 1.3). In this study, only 172 (40.7%) [95% CI: 36.3, 45.7] of the respondents were found to be knowledgeable about prevention of surgical site infection.

### 3.3. Factors Associated with Knowledge of Nurses about Prevention of SSI

In the bivariate analysis, age, service year, sex of the participants, and ever taking training on infection prevention methods were factors which were significantly associated with knowledge about prevention of surgical site infection. However, only service year, sex of the participants, and ever taking training on infection prevention were found to be significantly associated in the multivariate analysis.

Male nurses were about 3 times more likely to be knowledgeable about prevention of surgical site infection than female participants (AOR = 3.22, 95% CI: 2.09, 4.95). Those nurses who have served for more than 5 years were about 2 times more likely to be knowledgeable about prevention of surgical site infection than those whose service years are 5 years or less (AOR = 1.81, 95% CI: 1.12, 2.94). Those nurses who have ever taken training on infection prevention methods were about 2 times more likely to be knowledgeable about prevention of surgical site infection than those who have not (AOR = 1.95, 95% CI: 1.27, 2.99) (Table 2).

### 3.4. Practice of Nurses on Prevention of SSI

In this study, the proportion of nurses who had good practice of surgical site infection prevention activities was found to be 206 (48.7%).

### 3.5. Factors Associated with Practice of SSI Prevention Activities

In the bivariate analysis, age, service years, sex of the participants, ever taking training on infection prevention methods, and educational level were found to be significantly associated with practice of surgical site infection prevention activities. However, only age, sex, and educational level of the participants were found to be significantly associated in the multivariate analysis.

Female nurses were about 2 times more likely to practice surgical site infection prevention activities as compared to male nurses (AOR = 2.35, 95% CI: 1.58, 3.50). Those nurses who are 30 years or older were about 2 times more likely to practice surgical site infection prevention actives as compared to those who are less than 30 years old (AOR = 1.79, 95% CI: 1.08, 2.97). Nurses who have diploma were about 2 times more likely to practice surgical site infection prevention activities as compared to those who have B.S. degree or higher (AOR = 2.26, 95% CI: 1.08, 4.76) (Table 3).

## 4. Discussion

Prevention of SSIs is one of the most important challenges in delivering optimum nursing care. Although all health professionals involved in patient care are responsible for ensuring patient safety in this regard, nurses play a major role since they are usually involved in each step around the clock [19]. According to literature, nurses' role in the prevention of SSIs is very crucial [20]. Therefore, nurses must have adequate knowledge and good practice regarding the prevention of SSIs.

In this study, the proportion of nurses who were knowledgeable about prevention of surgical site infection was found to be 40.7% with a mean score of 56.3%. This finding indicated that more than half of the nurses working in the two referral hospitals demonstrated inadequate knowledge on prevention of surgical site infections, a finding in line with many of similar and related studies in Africa and western countries [13, 21, 22]. Similarly, this finding is in agreement with a Nigerian study in that only 40% of the participants had adequate knowledge regarding prevention of SSIs [16]. In the Nigerian study, the majority of nurses (66%) had adequate knowledge on general infection control mechanisms in contrast with the low proportion in the same participants regarding prevention of SSIs (40%). The implication of the Nigerian finding is that prevention of SSIs needs additional evidence-based knowledge apart from general information on infection control which might be acquired during college study. Therefore, since the knowledge-based questions were designed based on up-to-date guidelines, the possible reason for lower finding in the current Ethiopian study might be lack of in-service refreshment trainings on evidence-based SSIs prevention guidelines and recommendations.

This study revealed that sex of the study participants was significantly associated with knowledge about prevention of surgical site infection. Male nurses were found to be three times more likely to be knowledgeable about prevention of surgical site infection when compared with female nurses. The possible explanation of this finding might be linked with the educational level of male nurses in that in this study the majority of the B.S. or M.S. holders were male nurses. Therefore, the difference in knowledge score could be due to the difference in educational level as those participants who had B.S. or M.S. degree are more likely to have better knowledge than diploma holders.

Year of service was another sociodemographic factor which was significantly associated with knowledge about prevention of surgical site infection. Those study participants who have served for more than five years were about two times more likely to be knowledgeable about prevention of surgical site infection than those whose service years are five years or less. This finding is in line with findings from European and African studies in which year of experience was positively associated with knowledge regarding infection prevention [16, 21]. The positive association from this study could be due to the fact that as the number of years of practice increases, health workers are more likely to be exposed to surgical departments repeatedly and became more experienced through working with senior medical staff.

In this study, knowledge about prevention of surgical site infection was significantly associated with ever taking training on infection prevention methods. Those nurses who have ever taken training on infection prevention methods were about two times more likely to be knowledgeable about prevention of surgical site infection than those who do not. This finding is comparable with a result across the literature where training of staffs on safe practices was positively associated with the knowledge about health care associated infections [13, 19]. This could be due to the fact that updating the knowledge of the health workers about prevention of infection could have changed the older understanding of the health workers and could have resulted in good score on knowledge questions. Moreover, since the current infection prevention and patient safety national guideline of Ethiopia incorporated detailed information and evidence-based recommendations about prevention of surgical site infection, nurses who have taken this training could have better knowledge regarding prevention of SSIs.

This study has also tried to identify the level of practice of the nurses in the study areas regarding prevention of surgical site infection. In this study, the proportion of nurses who were practicing proper surgical site infection prevention activities was 48.7% (95% CI: 43.9, 53.5). This result is lower than the result from a study done in an Egyptian hospital where 57.1% of the health workers were found to practice infection prevention activities satisfactorily. However, it is higher than findings from studies done in Italy where only 38% of the nurses practice use of all infection prevention methods. This could be due to difference in attitude of the health workers towards applying infection prevention methods. It could also be due to the difference in the operational definition of the satisfactory practice from study to study. Difference in knowledge of the health workers concerning prevention of surgical site infection could also be another factor for this discrepancy.

Findings from this study showed that age of the study participants was one of the sociodemographic factors which were significantly associated with the practice of activities of prevention of surgical site infection. This study showed that nurses who are 30 years or older were about two times more likely to practice surgical site infection prevention activities properly when compared with those who are less than 30 years old. Positive association of year of service with practice of infection prevention activities can be explained by the fact that practice makes perfect, which means they might have improved their practice from year to year.

Sex of the nurses was another factor which was significantly associated with good practice of activities of surgical site infection. Female nurses were about two times more likely to practice surgical site infection prevention activities when compared with male nurses.

The other factor which was significantly associated with the good practice of surgical site infection prevention activities is educational status of the study participants. In contrast to many other studies, diploma nurses were about two times more likely to practice surgical site infection prevention activities when compared to those with B.S. degree or higher. This negative finding might be partly due to the educational system of the nursing schools where in all three-year diploma nursing programs the percentage of practical courses is 70% with 30% theoretical supplement; however, in a four-year degree program the percentage of practical courses is below 50%.

## 5. Conclusion

Knowledge and practice of surgical site infection prevention activities among nurses working in Gondar and Debre Markos Referral Hospitals were found to be low. Being male, serving for more than five years, and taking training on infection prevention activities were factors which were significantly associated with knowledge of prevention of surgical site infection. On the other hand, being thirty years or older, being female, and being of diploma level were factors which were significantly associated with good practice of surgical site infection prevention activities. Therefore, efforts have to be made to update the knowledge of nurses regarding surgical site infection prevention activities. Moreover, hospital administrators should encourage highly educated nurses to focus on implementing their knowledge into practice.

## Figures and Tables

**Table 1:** Sociodemographic characteristics of the study participants, Gondar and Debre Markos Referral Hospitals, Northwest Ethiopia, 2015 (*n* = 423).

Variable	Frequency	Percent
Age		
20–29 years	340	80.4
≥30 years	83	19.6
Sex		
Male	239	56.5
Female	184	43.5
Marital status		
Single	244	57.7
Married	179	42.3
Educational level		
Diploma	36	8.5
B.S. degree	379	89.6
Master's degree	8	1.9
Ward		
Surgical	157	37.1
OB/GYN^*∗*^	74	17.5
Medical	83	19.6
Pediatrics	63	15.0
OPD^*∗∗*^	46	10.8
Ever took infection prevention training		
Yes	188	44.4
No	235	55.6

^*∗*^OB/GYN: obstetrics and gynecology; ^*∗∗*^OPD: outpatient department.

**Table 2:** Multivariate logistic regression of factors associated with knowledge about surgical site infection prevention activities among nurses working in Gondar and Debre Markos Referral Hospitals, Northwest Ethiopia, 2015 (*n* = 423).

Variables	Knowledgeable		OR (95% CI)	
	Yes	No	COR (95% CI)	AOR (95% CI)
Sex				
Male	123 (51.5%)	116 (48.5%)	2.92 (1.93, 4.42)	**3.22** (**2.09**, **4.95**)
Female	49 (26.6%)	135 (73.4%)	1	1
Age				
≥30 years	43 (51.8%)	40 (48.2%)	1.76 (1.09, 2.85)	*∗*
<30 years	129 (37.9%)	211 (62.1%)	1	
Service years				
More than 5 years	58 (52.7%)	52 (47.3%)	1.95 (1.25, 3.02)	**1.81** (**1.12**, **2.94**)
Five years or less	114 (36.4%)	199 (63.6%)	1	1
Ever took IP training				
Yes	95 (50.5%)	93 (49.5%)	2.10 (1.41, 3.11)	**1.95** (**1.27**, **2.99**)
No	77 (32.8%)	158 (67.2%)	1	1

^*∗*^Not significant.

**Table 3:** Multivariate logistic regression of factors associated with practice of nurses in surgical site infection prevention activities in Gondar and Debre Markos Referral Hospitals, Northwest Ethiopia, 2015 (*n* = 423).

Variables	Good practice		OR (95% CI)	
	Yes	No	COR (95% CI)	AOR (95% CI)
Sex				
Female	111	73	2.31 (1.56, 3.41)	**2.35** (**1.58**, **3.50**)
Male	95	144	1	1
Age				
≥30 years	51	32	1.90 (1.16, 3.11)	**1.79** (**1.08**, **2.97**)
<30 years	155	185	1	1
Service years				
More than 5 years	60	50	1.37 (0.09, 2.12)	*∗*
Five years or less	146	167	1	
Ever took IP training				
Yes	99	89	1.33 (0.91, 1.96)	*∗*
No	107	128	1	
Educational level				
Diploma	24	12	2.25 (1.10, 4.63)	**2.25** (**1.08**, **4.76**)
B.S. degree + M.S.	182	205	1	1

^*∗*^Not significant.
